# Data Mining Approaches for Genomic Biomarker Development: Applications Using Drug Screening Data from the Cancer Genome Project and the Cancer Cell Line Encyclopedia

**DOI:** 10.1371/journal.pone.0127433

**Published:** 2015-07-01

**Authors:** David G. Covell

**Affiliations:** Screening Technologies Branch, Developmental Therapeutics Program, National Cancer Institute, Frederick, Maryland, United States of America; Institute of Cancer Research (ICR), UNITED KINGDOM

## Abstract

Developing reliable biomarkers of tumor cell drug sensitivity and resistance can guide hypothesis-driven basic science research and influence pre-therapy clinical decisions. A popular strategy for developing biomarkers uses characterizations of human tumor samples against a range of cancer drug responses that correlate with genomic change; developed largely from the efforts of the Cancer Cell Line Encyclopedia (CCLE) and Sanger Cancer Genome Project (CGP). The purpose of this study is to provide an independent analysis of this data that aims to vet existing and add novel perspectives to biomarker discoveries and applications. Existing and alternative data mining and statistical methods will be used to a) evaluate drug responses of compounds with similar mechanism of action (MOA), b) examine measures of gene expression (GE), copy number (CN) and mutation status (MUT) biomarkers, combined with gene set enrichment analysis (GSEA), for hypothesizing biological processes important for drug response, c) conduct global comparisons of GE, CN and MUT as biomarkers across all drugs screened in the CGP dataset, and d) assess the positive predictive power of CGP-derived GE biomarkers as predictors of drug response in CCLE tumor cells. The perspectives derived from individual and global examinations of GEs, MUTs and CNs confirm existing and reveal unique and shared roles for these biomarkers in tumor cell drug sensitivity and resistance. Applications of CGP-derived genomic biomarkers to predict the drug response of CCLE tumor cells finds a highly significant ROC, with a positive predictive power of 0.78. The results of this study expand the available data mining and analysis methods for genomic biomarker development and provide additional support for using biomarkers to guide hypothesis-driven basic science research and pre-therapy clinical decisions.

## Introduction

Large-scale sequencing efforts, headed mostly by the International Cancer Genome Consortium (https://icgc.org/) and The Cancer Genome Atlas (http://cancergenome.nih.gov/), have contributed to the development of drug treatments that selectively target genomic changes; as for example; BCR-ABL1 translocations (Imatinib)[1,2], EML4-ALK translocations (EGFR and ALK inhibitors) [3] and BRAF:V600E mutation(BRAF inhibitors)[4]. More recently, efforts to systematically identify genomic changes that might serve as biomarkers of therapeutic drug susceptibility have led to collaborations between The Wellcome Trust Sanger Institute and Massachusetts General Hospital (data for more than 700 immortalized tumor cells and 138 cancer drugs) and the Broad Institute and Novartis collaboration (profiling 24 cancer drugs across 479 immortalized tumor cells); each effort guided, in part, by the pioneering NCI60 drug screen [5]. Although critics of these efforts often note limitations of immortalized human tumor cells to account appropriately for tumor-stroma interactions, immune surveillance, invasion and metastasis, angiogenesis and the role of stem cell populations[6], proponents are testing whether genomic biomarkers derived from these screens can be used reliably to assist hypothesis-driven basic science efforts, and clinical efforts to assign therapy, monitor response and predict outcomes (e.g. Precision Medicine, MATCH Trial, IMPACT, I-SPY). As the pipeline of new drug discoveries expands, progress towards achieving more effective treatments may be aided by research efforts that vet existing, as well as develop new methods for identifying genomic biomarkers that are associated with compound efficacy.

## Background

The CGP[7] and CCLE[8] reports offer compelling associations between drug sensitivity (typically measured by the log of the drug concentration for 50% growth inhibition, referred to throughout the text as GI50) and specific genomic changes, inclusive of gene expression (GE), gene mutation (MUT), copy number (CN), and translocations. Their results find advantages of multi-gene, versus single-gene biomarkers, as indicators of tumor cell GI50; stemming, at one extreme, from failures to find reliable associations between a single gene’s changes and GI50; and, at the other extreme, from instances where GI50 appears to be mediated by diverse, somewhat unconnected, multi-gene, biological mechanisms. Moreover, their expert application of state-of-the-art data mining and statistical methods represents a systematic approach that yielded results consistent with drug-sensitizing translocations and MUTs known to be predictive of clinical outcomes. Collectively these efforts represent a crucial step in gaining an understanding of cancer, based on the genomic characterization of human tumor samples against a range of cancer drug responses that correlate with genomic change. As these and other systematic efforts continue, it is important to recognize that public access to the CGP and CCLE data provides a rich and unique opportunity for independent assessments of this data[9] that might contribute to the further development of multi-featured genomic biomarkers as guides to basic and pre-clinical research and early clinical trials. Motivated by these goals, and building from these previous efforts, this analysis will focus on i) vetting existing results, ii) using alternative data mining and statistical methods for biomarker discovery, iii) providing novel interpretations of the CGP and CCLE databases and iv) assessing the use of biomarkers as predictive of tumor cell drug response.

## Methods

Data mining and statistical strategies applied to the analysis of large databases are often comprised of standard and user-defined (ad hoc) components which can play pivotal roles in data interpretation. The data mining and statistical strategies applied here share many similarities with those used in Garnett et al.[7] and Barretina et al.[8]: inclusive of hierarchical clustering, Elastic Net (EN) regression and pathway analysis of selected genes. Noteworthy departures include; i) modifications of their method for hierarchical clustering of GI50 values, ii) applications of EN regressions based solely on GEs, iii) followed by assessments of roles of CN and MUT in GI50 responses, iv) extensions of EN gene sets to include Gene Set Enrichment Analysis (GSEA) to hypothesize biological pathways contributing to GI50 responses, v) applying a global analysis of GE, CN and MUT data using a false discovery rate (FDR)-adjusted selection of significant associations of these biomarkers with drug response and vi) applications of ROC analysis for CGP-derived genomic biomarkers as predictors of GI50 in the CCLE data. Brief descriptions of these alternative methods and will be discussed below. More detailed information appears in S1 File.

### Hierarchical Clustering of GI50

Absence of similar GI50 values for drugs having the same mechanism of action (MOA) presents a major hurdle for attempts to associate genomic signatures with drug response; and extend these associations to hypothesize biological processes having roles in drug efficacy. As noted in the CGP report[7], drugs with overlapping specificity (referred to hereafter as a MOA class) did not always share correlated GI50 values, nor did they always share genomic signatures. The hierarchical cluster analysis of Garnett et al.[7] classified drugs into *clusters* based on GI50 similarity, with intra-cluster drug correlations, yielding 22 *community clusters*, using measures of drug sensitivity for the ~700 tumor cells in the CGP data Supplementary Table 1[7]). While the intention of the effort here does not seek to exhaustively sample available hierarchical clustering methods and schemes to identify *community clusters*, a reasonably good association between GI50 values for drugs of a similar MOA class could be found with slight modifications in the clustering methodology of Garnett et al.[7]. Rather than hierarchical clustering based on GI50 similarity, all pairwise GI50 correlations were used for each drug and randomized resampling [10] was used to determine *community clusters*. This clustering procedure, available as the CRAN installed package, pvclust[10], in the R programming language, considers the importance of sampling error’s contribution to uncertainty in cluster results by using a randomized resampling method to identify cases that have a high frequency of occurring as cluster members. Additional details of this cluster analysis appear in **S1 File: A. Hierarchical Clustering of GI50, Fig A, Fig B** and **C. Concordance between MOA class and EN GE**.

### Elastic Net Regression of Gene Expression

**Fig 1:**
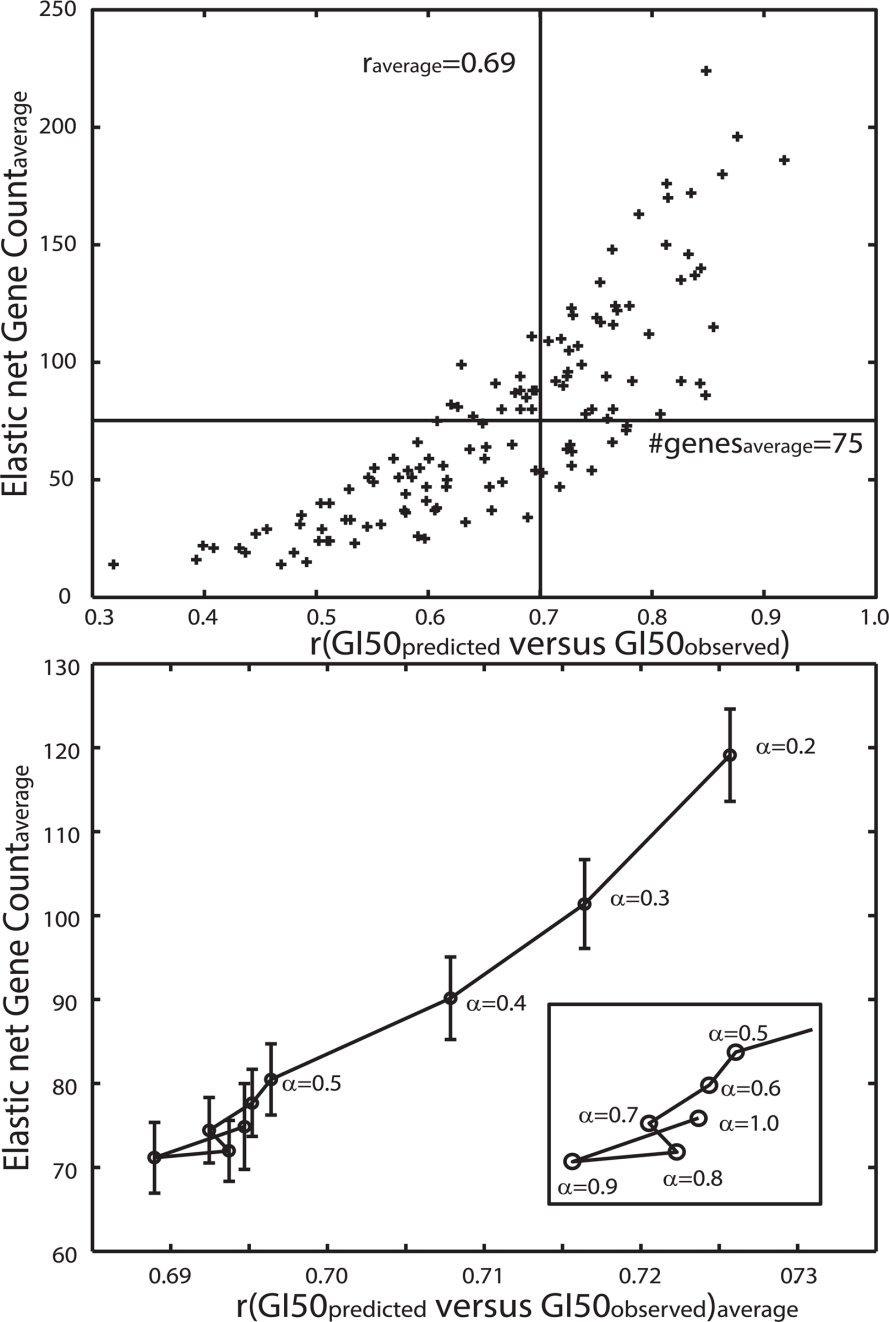
Upper panel. **Plot of correlation coefficient (GI50**_**predicted**_ **against GI50**_**observed**_ **) versus number of genes in the converged EN regression model for α = 0.7.** These results yield an average correlation of 0.69 (±0.12) between GI50_**observed**_ and GI50_**predicted**_ with a mean number of 75(±44) EN gene expressions for 129 drugs where the EN regression converged. Lower panel. Plot of the average correlation between EN model fits versus their average number of EN genes. Results representα ranging from 0.2 to 1.0. Error bars represent standard error of the mean. Boxed region in the lower right displays results for α > = 0.5).

Elastic Net (EN) regression is a statistical procedure that fits a generalized linear model of observations (genomic data) to GI50 values across a set of tumor cell. As an alternative to grouping all the genomic data (GE, CN and MUT) into the EN analysis[7], the results here will focus on EN analysis derived only from GEs measured in the CGP tumor cells; followed by assessments of CN and MUT status for these EN-derived genes. EN analysis has been completed using the glmnet package[11] in the R-programming language. Glmnet provides an adjustable parameter, α, that allows EN regression to range from a lasso (α = 1) to a ridge (α = 0) regression. The latter application typically generates an exact fit (GI50_predicted_) of the GI50 data (GI50_observed_) using all of the GEs for all tumor cells with a GI50 measurement, while the lasso instance models GI50 using a reduced set of GEs to yield a less than perfect fit between GI50_predicted_ and GI50_observed_. Clearly, a model that fits GI50 perfectly, while using all of GEs, provides no reduction in the numbers of genes for assessing their potential role as biomarkers for a compound’s GI50. Selecting an appropriate balance between the goodness of the EN model’s fit to the data and the numbers of genes selected in the EN regression can be determined from results obtained at different values of α. For example, the upper panel in **Fig 1** plots the correlation coefficient (GI50_observed_ and GI50_predicted_) versus EN gene count for α = 0.7. Evident from this result is the tendency to obtain a better EN model fit with larger numbers of EN genes. Conversely, EN results that use only a few genes appear to have a reduced capacity to accurately predict GI50. The lower panel in **Fig 1** plots the average correlation coefficient versus the average number of EN genes (with the EN regression converging for 129 of the 138 drugs) for α ranging from 0.2 to 1.0. As expected, the trend of better EN model fits using larger numbers of genes (lower values of α) is evident. This result finds a general grouping at the lower left corner forα in the range of 0.6 to 1.0. Using this result, a heuristic selection of α = 0.7 was chosen as a reasonable balance between goodness of EN fit and reduced numbers of EN genes. Choosing α = 0.7 yielded an overall fitting accuracy of ~0.5 (r^2^), using an average of 75 EN genes. Analyses based on slightly lower or higher choices for α did not appear to strongly influence the results to follow.

**Fig 2:**
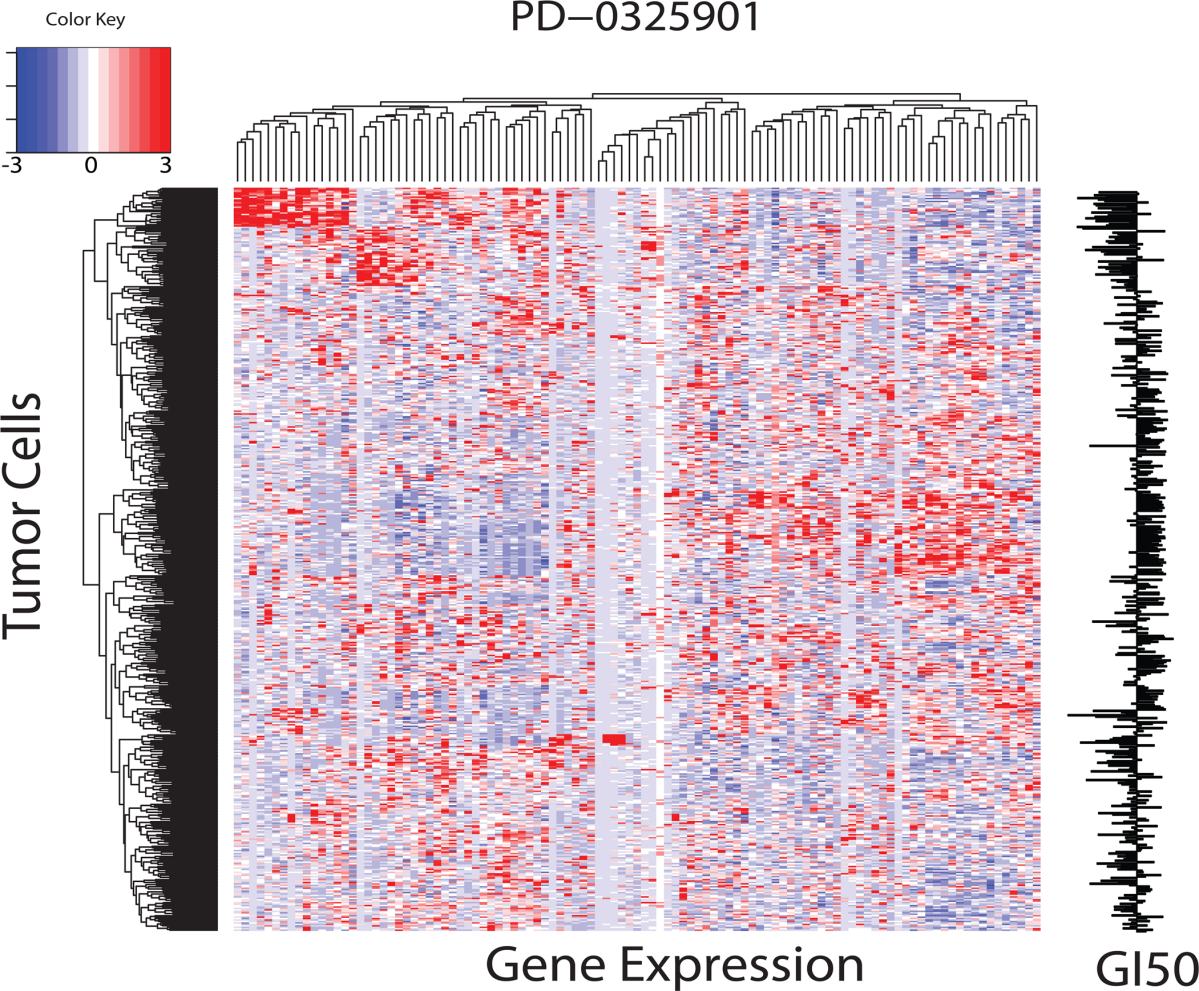
Heatmap of EN model (alpha = 0.7) for PD-0325901 (a MEK1/2 targeting compound). Figure plots the 103 gene expressions (x-axis) for the 514 tumor cells of EN model (y-axis). Results depict only tumor cells having a GI50 measurement against PD-0325901.Heatmap is ordered along each axis according to the dendrograms displayed at the top and left edge. Over and under expressed genes are indicated by red and blue colors, respectively. GI50_**observed**_ for these 514 tumor cells appears as a bar graph at the right edge of the image. Bars to the left and right correspond to sensitive and insensitive GI50 responses, respectively.

A typical output from the glmnet calculation, using the example of PD-0325901 (a MEK1/2 targeting compound), appears in **Fig C 3.** This figure displays the EN gene count versus the model Mean-Squared Error (MSE). For this example, the model reached a minimum MSE using 103 genes, representing a reduction of 99.2% from the 13,325 gene expressions within the set of 514 tumor cells having a GI50 response to PD-0325901. EN regression yields a correlation of 0.84 between GI50_observed_ and GI50_predicted_. **Fig 2** displays the heatmap (using heatmap.2 in the R programming language) for the 103 gene expressions across 514 tumor cells for PD-0325901. The rightmost edge of this image displays a barplot for GI50_observed_ for these 514 tumor cells. Patchwork blocks of red and blue in the heatmap represent relatively over and under expressed genes, respectively, exhibiting a qualitative association of these GE patterns with the barplot of GI50 for each tumor cell displayed at the left edge. Heatmaps of EN GEs will be used, qualitatively, for visual comparisons of over and under expressed genes associated with drug sensitivity and insensitivity.

### Concordance between MOA class and EN GE

EN regression genes can be subjected to a hierarchical cluster analysis to assess concordance between drugs of similar MOA classes and their EN gene expressions (used to model GI50). Concordance will be measured by determining whether a) EN genes appear as cluster neighbors for drugs (i.e. MOA classes) and b) whether these EN genes are relatively unique to each MOA class. Answering part a) will establish whether MOA concordance based on similarity in GI50_observed_ also exists when using the expression of EN genes used to model GI50_observed_. Answering part b) is pivotal for developing gene expressions as biomarkers of GI50 response to specific MOA classes of drugs and extending these results to hypothesize biological pathways involved in drug efficacy. A more detailed description of this analysis appears in **S1 File; C. Concordance between MOA class and EN GE:)**

### GSEA analysis of EN derived GEs

**Fig 3:**
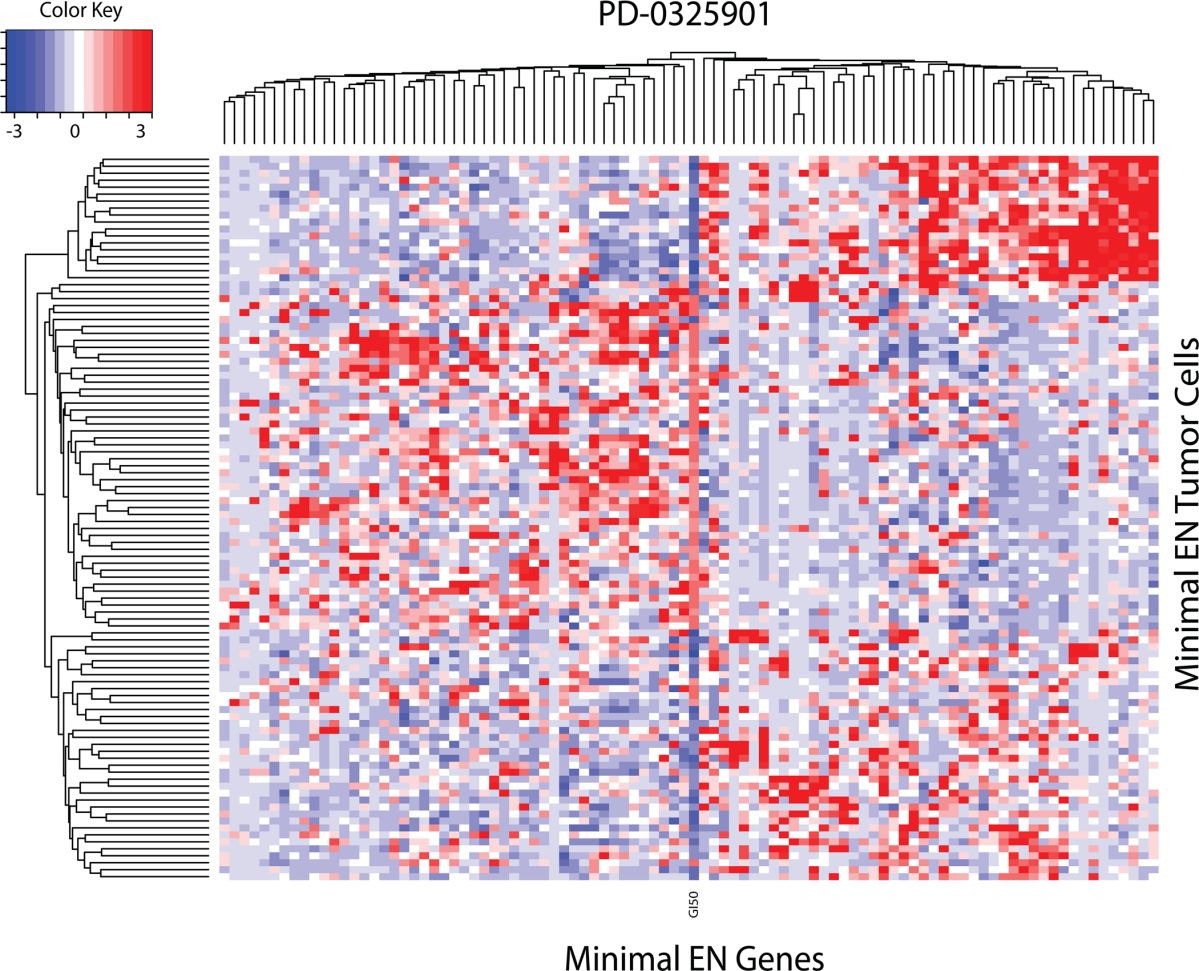
Heatmap for minimal EN gene expressions (x-axis) versus the upper and lower 10^th^ percentile of sensitive and resistant tumor cells (y-axis) for PD-0325901. Relative over and under expression is denoted by red and blue, respectively. GI50 values for PD-0325901 are imbedded in the heatmap, located as the column near the middle, labelled as GI50. GI50 values for sensitive and resistant tumor cells are indicated by blue and red colors, respectively.

Following Garnett et al.[7], examination of EN genes for the most and least sensitive tumor cells can be used to prioritize GI50-EN gene associations. Towards this end, the EN genes for each drug were filtered by conducting a Student’s t-test to identify statistically significant (p<0.05) EN GEs between the top most and bottom least 10^th^ percentile of tumor cell drug responses (this model will be referred to hereafter as the ‘minimal EN model’ for each drug). **Fig 3** displays the heatmap for the minimal EN model of the PD-0325901 example shown in **Fig 2.** Rather than display GI50 as a bar graph at the edge (as in **Fig 2**), the GI50 data is embedded into the heatmap (see column labelled ‘GI50’ located near the center of the image), where the most sensitive cells, identified in dark blue, appear in the upper and lower portions of the heatmap and the most resistant cells, with their GI50 values identified in red, appear in the middle portion of the heatmap. In this example, an overall reduction of 82% (1-94/514) in tumor cell count and a 11% reduction (103 down to 94 genes) in PD-0325901’s EN gene set remain in its minimal EN model.

EN regression represents a means to identify a reduced set of genes whose expressions are sufficient to yield a reasonable model of each drug’s GI50 response (cf. **Fig 1**) and can be used to hypothesize biological pathways that might play a role in a drug’s response. Many computational tools currently exist for pathway analysis (GSEA, DAVID, Ingenuity, etc.). Included in cautionary warnings for these methods is that results can lead to over interpretations, when genes are shared between many pathways, or yield no information, for cases either lacking statistically significant pathways or where large numbers of pathways are found that do not reveal a consistent biological theme. GSEA[12] offers a heuristic hedge against these warnings by limiting results to only pathways with at least 2 shared genes and applying a False Discovery Rate (FDR) against a chance finding at the typical threshold of 0.05. The former requirement avoids instances of large numbers of pathways with only one EN gene, while the latter requirement limits the chance occurrence of pathways with many shared, and frequently occurring, genes. Based on these considerations, GSEA, using minimal EN-derived GEs, was used to hypothesize biological processes that might be related to drug response. GSEA reporting will be restricted to only the topmost significant (FDR score) pathways, limited to no more than 10 cases.

Reporting of GSEA results will emphasize recurrent biological themes for significant pathways rather than individual pathways. As an illustration, GSEA[12] pathways, derived from the KEGG, BIOCARTA and GO gene subsets, using the minimal EN GEs for the MEK inhibitor, PD-0325901, are listed in **Table C.** These results find DNA_REPAIR as the GSEA pathway with the best statistical significance, with RESPONSE_TO_DNA_DAMAGE_STIMULUS and DNA_METABOLIC_PROCESS as the next most significant pathways. Further down the list are three pathways related to SIGNALLING. The general themes of these GSEA results indicate that the tumor cell response to PD-0325901 would be hypothesized to involve DNA and SIGNALLING. Evidence of an association between MEK-ERK signaling and DNA_REPAIR has been reported by Sato et al.[13] and Marampon et al.[14], leading to the proposal of using MEK inhibitors to increase tumor cell radiosensitivity by down regulating DNA repair signals. More recently Pei et al. [15] have proposed a combination therapy for multiple myeloma using a CHK1 inhibitor to prevent cells from arresting in stages of the cell cycle that facilitate the repair of DNA damage and a MEK inhibitor to prevent cells from activating a variety of proteins that regulate DNA repair processes while promoting the accumulation of pro-death proteins. The GSEA findings here, of pathway themes related to DNA repair or damage and cell signalling, are consistent with hypothesizing a role of PD-0352901 in signals related to DNA maintenance.

Considerable caution must be applied when interpreting these results. For example, although the other three MEK1/2 inhibitors, CI-1040, AZD6244 and RDEA199, appear within the same cluster, based on GI50 (**Table A**) and EN GEs (**Table B** and **Fig D**), only AZD6244 shares some of its GSEA pathways with PD-0325901, while CI-1040 and RDEA119 do not. Collectively, these results, while supporting a general consistency within these MEK1/2 inhibitor’s GI50 profiles (**Table A**), with a sufficiently unique set of EN genes for them to appear within common clusters (**Table B** and **Fig D**), yield EN genes sufficiently different from each other to generate non-overlapping GSEA pathways. A plausible factor contributing to these EN-gene GSEA differences may be cellular potency, where PD-0325901 is, on average, more than an order of magnitude more potent than the other three MEK1/2 inhibitors for the CGP tumor cells. Apparently the EN genes for PD-0325901 are sufficiently unique to reveal its role in DNA maintenance and signaling not found for the other MEK1/2 inhibitors. These results emphasize the likelihood that although compounds may share a putative MOA target and generate similar GI50 responses, GSEA of minimal EN genes represents only a hypothetical association between unique sets of EN genes and specific biological processes related to each drug’s GI50. While existing literature support will be provided for GSEA selected pathways, biological confirmation will clearly be required.

### Global analysis of CN and MUT for minimal EN GEs

The results for individual drugs can be extended to include a global analysis of the CGP data describing MUTs and CN changes that potentially play a role in drug response. Analogous to the earlier analysis, where minimal EN genes were identified based on having a statistically significant difference in GE between the most and least sensitive tumor cells, significant gene MUTs and CN changes can be determined in an identical manner. Selecting each drug’s minimal EN tumor cells, a two-tailed Student’s t-test was used to calculate all the p-values based on MUT or CN differences between the most sensitive and resistant tumor cells. These results were filtered by using a Benjamini-Hochberg (B-H)[16] false discovery rate of 0.1 to identify significantly different biomarkers. The t-statistic for these comparisons provides a convenient measure for hierarchical clustering of significant results. Heatmap visualizations can be color-coded from blue to red to indicate the strength of statistical significance, where the red portion of the spectrum reflects cases where the resistant tumor cells exhibit higher biomarker responses when compared to the sensitive tumor cells and the blue portion of the spectrum represents the case of higher biomarker values in the sensitive tumor cells when compared to the resistant tumor cells. The numbers of significant MUTs are sufficiently small to associate subsets of genes to specific GSEA pathways. In contrast, the numbers of genes with significant CN changes are sufficiently large to require further hierarchical clustering of GSEA pathways for ease of interpretation.

### GSEA analysis of significant MUTs and CNs

Heatmap visualizations of the statistically significant MUTs and CNs that pass the BH-adjusted threshold for statistical significance can be used for a globally-based GSEA. The cluster dendrograms of significant CN and MUTs can be cut to yield small groups of genes for GSEA. These results generate a globally-derived FDR-adjusted significance score for biological pathways associated with sub-clusters of minimal EN GEs. Clustering of these globally-derived scores can be used to associate GI50 responses with biomarkers having statistical significance between resistant and sensitive tumor cell responses.

### ROC analysis of CGP GEs as predictive of CCLE drug response

‘Signature’ genes are commonly used to assess whether a subset of gene expressions are sufficiently comparable to indicate a likelihood of a similar biological condition or therapeutic response [17,18]. Minimal EN GEs may also be proposed as signature genes for predicting drug response. In order to test this premise, the minimal EN GEs developed for the CGP set of drugs were used to select for non-CGP tumor cells with matching GEs as predictors of drug efficacy for test drugs. Failure to achieve any success with this method could influence future applications of this approach. However, moderate success may offer motivation for devising more optimal steps for achieving favorable outcomes with this approach. The CCLE dataset (24 drugs tested against 479 tumor cells) shares 16 drugs with the CGP datasets. Using the CGP-derived minimal EN model for each of the 16 matching drugs, GEs between these two datasets will be compared (using their mean squared error, MSE) and used to rank the complete set of CCLE’s tumor cells. In order for the ‘test’ biomarker to have predictive utility, MSE scores must correctly rank a CCLE tumor cell’s drug response within the top (sensitive) or bottom (resistant) of all CCLE tumor cells. Only the top 5^th^ percentile of MSE scores for the CCLE tumor cells will be selected. It is noteworthy to re-emphasize that the minimal EN model uses GEs to predict GI50. Thus sensitivity and resistance are integral parts of this model. Standard assessment of false/true-positives/negatives using ROCS will be used to evaluate results.

## Results

### Hierarchical clustering of GI50

The concordance between drugs of the same MOA class and GI50 finds reasonably good agreement. Using a modified hierarchical clustering (pvclust) and a modified metric (all-to-all correlations of GI50), over half (16/30 = 0.53) of the drugs that share a MOA class also appear within the same community cluster; with 4 of the 5 SRC agents common to one cluster. This analysis was extended to determine the concordance between MOA and co-clustering of EN-derived GEs (see **S1 File. - C. Concordance between MOA class and EN GE** for more details). Filtering the 129 drug’s EN regressions that converged and yielded greater than 10 EN genes yielded fewer than ~2k of the original 13,325 GEs for the 87 drugs that share at least 2 EN genes. Hierarchical clustering of the gene expression for these filtered genes (**Fig D**) finds that greater than two-thirds (59/87 = 0.68) of the EN gene expressions for drugs with a shared MOA appear in the same cluster. These results indicate that hierarchical clustering, based on GEs derived from EN-regression models of GI50, yields a higher concordance within MOA drug classes when compared to clustering based on GI50 similarity alone. The average overlap of only 1.67 between EN-genes for each drug suggests that EN-genes are relatively unique for each drug. Collectively, the relatively high concordance, using either GI50 or EN-derived GEs that model GI50, and the existence of relatively few shared genes in each drug’s EN model, support the potential application of gene-based measures as unique biomarkers for GI50.

### Minimal EN regression

Each drug’s minimal EN model yields a reduced set of genes that may play a role in its GI50. Following the report of Garnett et al.[7], the minimal EN GEs, CNs and MUTs with the greatest statistical significance between sensitive and insensitive tumor cell response can be examined for consistency with literature reports, as well as hypothesizing novel biological roles in each drug’s response. Results for selected compounds will be reported.

#### Cisplatin

The first example, using the DNA cross-linker, cisplatin, confirms the results of Garnett et al.[7] Seventy EN genes and 108 tumor cells define its minimal EN model. Statistical analysis of significant differences in CN and MUT status of only the minimal EN genes for the cisplatin sensitive and resistant tumor cells (listed in **Table 1**) finds that sensitivity to cisplatin is associated with MUTs in EWS_FLI1, PTEN, ERBB2 and APC (http://cancer.CGP.ac.uk/CGP/gene/overview?ln=APC and Niedner et al.[19]). Not included in the CGP report [7] is the appearance of KRAS_MUT as a potential biomarker of cisplatin sensitivity. Support for this additional perspective appears recently in Lin et al.[20], where KRAS_MUT was found to be a predictor of sensitivity to the cisplatin analog oxaliplatin. KRAS overexpression by mutant vectors caused excision repair cross-complementation group 1 (ERCC1) down regulation in protein and mRNA levels, and enhanced oxaliplatin sensitivity. The importance of XRCC1 in cisplatin sensitivity is further supported by Xu et al.[21] where the protein expression of XRCC1 was significantly increased in cisplatin-resistant cells and independently contributed to cisplatin resistance. The results in **Table 1** also extend the cisplatin analysis to hypothesize roles in cisplatin sensitivity for CN changes of two histone lysine demethylases (KMD6A_CN and KMD5C.JARDIC_CN). Epigenetic roles of histone lysine demethylases are beginning to emerge as important in breast and ovarian cancers [22].

**Table 1:** Genes with the most statistically significant differences between MUT or CN values in sensitive and resistant tumor cells using the minimal EN model of cisplatin.

MUT	p-value
EWS_FLI1_MUT	0.006181
PTEN_MUT	0.007763
ERBB2_MUT	0.011771
KRAS_MUT	0.012336
APC_MUT	0.022302
CN	
KDM5C.JARID1C_CN	0.005804
SSX2_CN	0.006626
KDM6A_CN	0.023417

#### Bortezomib

The minimal EN regression model for bortezomib consists of 44 genes and 64 tumor cells (**Fig E**), which modeled GI50_observed_ with a correlation coefficient of 0.69. Statistical results for the top most significant differentially expressed minimal EN genes between sensitive and insensitive tumor cells are listed in **Table 2**. The appearance of NQO2 at the top of this list may offer exploitable information about bortezomib therapy. NQO2 is a flavoprotein, functioning as a quinone oxidoreductase, known to protect cells against radiation and chemical induced oxidative stress. The 20S proteasome and NQO2 both interact with myeloid differentiation factor C/EBPalpha [23]. Another quinone oxidoreductase, NQO1, was found by CCLE [8] to be the top predictor of sensitivity to the Hsp90 inhibitor 17-AAG. Hsp90 plays a role in the assembly and maintenance of the proteasome [24]. Simultaneous inhibition of Hsp90 and the proteasome enhances antitumor activity of both drugs[25]. Although the exact mechanism for this observation is not yet resolved, the result presented here suggests a dual role for quinone oxidoreductase biomarkers (NQO2, NQO1) in the use of HSP90/proteasome targeting agents as single and combined therapies[25].

**Table 2:** GEs with the greatest statistical significance for comparisons between the sensitive and resistant tumor cells from the minimal EN model of bortezomib.

Gene	p-value
NQO2	6.41E-06
HTRA1	1.20E-05
TLR3	1.82E-05
EGR1	1.86E-05
PARVA	2.35E-05
PRKAG1	3.26E-05
EGR3	6.30E-05
ITM2B	1.40E-04
C10ORF56	1.75E-04
HEAB	4.29E-04

#### Temsirolimus

The next example, for the mTOR targeting agent temsirolimus, yielded a minimal EN model consisting of 67 genes and 108 tumor cells. A Student’s t-test for CN differences between the sensitive and resistant tumor cells of temsirolimus’ minimal EN model finds the most significant difference (p<0.00044) for the DNA helicase protein, WRN. Diseases affected by abnormal ribosome biogenesis via modulation of components which impact on Pol I transcription [26] include Blooms and Werner Syndrome [27]. There have been numerous clinically approved drugs whose therapeutic affect is mediated, at least in part, through disrupting ribosome biogenesis, including actinomycin D, cisplatin, irinotecan/topotecan, mitomycin C, 5-fluorouracil and temsirolimus [26]. In the case of temsirolimus, rRNA synthesis is inhibited by interfering with mTORC1 activity [26]. The results here find that an increased CN for WRN corresponds to enhanced temsirolimus sensitivity and may provide a biomarker for sensitivity to temsirolimus. Previous studies by Zoppoli et al.[28] and CCLE[8] have found that another DNA/RNA helicase, Schlafen-11 (SLFN11), sensitizes cancer cells to DNA-damaging agents. These results suggest an expanded role for DNA helicases in drug sensitization and their potential use as predictors of drug effects for agents targeting ribosome biogenesis.

No genes are shared between the minimal EN model for the mTOR targeting agents, rapamycin and temsirolimus. While this appears to be a surprising result, given that both agents have the same putative target, none of their minimal EN model tumor cells are common to both drugs. In addition, none of the CN data for the rapamycin resistant versus sensitive tumor cells achieve statistical significance, while three MUTs have relatively weak statistical significance (CDKN2a_MUT; p = 0.031, FBXW7_MUT; p = 0.048 and MYCN_MUT; p = 0.025). Collectively, these results add further support to the earlier finding with MEK1/2 inhibitors that each drug generates a relatively unique minimal EN regression model, both in terms of its genes, sensitive and resistant tumor cells and roles for metadata (e.g. CN and MUT) in drug efficacy.

#### ABT-888 and Dasatinib

**Fig 4:**
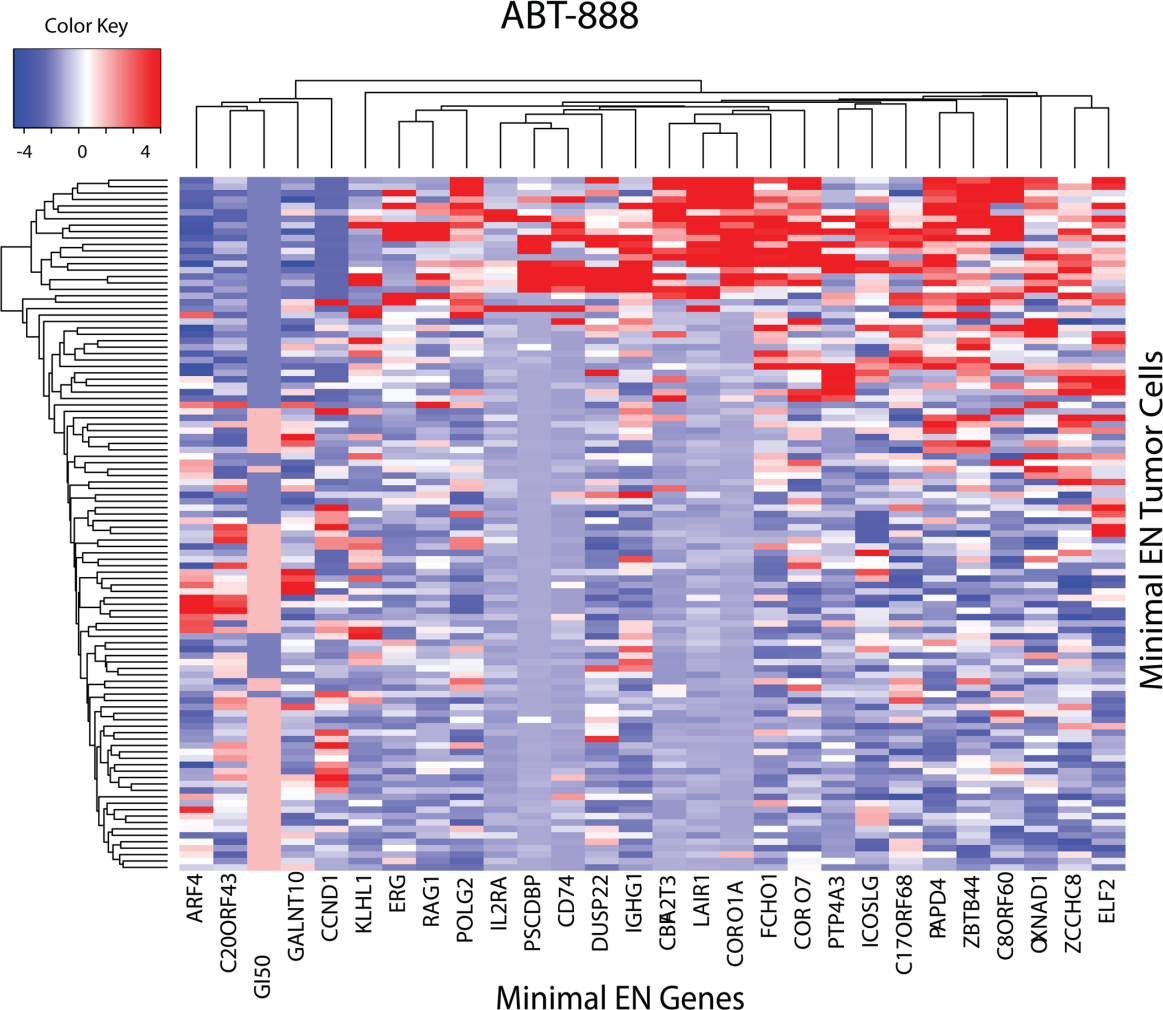
Heatmap for minimal EN GEs (x-axis) versus the upper and lower 10^th^ percentile of sensitive and resistant tumor cells (y-axis) for ABT-888. Relative over and under expression is denoted by red and blue, respectively. GI50 values for ABT-888 are embedded in the heatmap, located as the third column from the left, with its label offset towards the bottom from the EN gene labels. GI50 values for sensitive and resistant tumor cells are indicated by blue and red colors, respectively. Relative ARF4 GE appears as the first column from the left.

The next two examples will continue to focus on combining GSEA with heatmap results using minimal EN GEs. The heatmap of the minimal EN genes for ABT-888 is displayed in **Fig 4**. The GI50 values for the 108 tumor cells appear as the 3^rd^ column from the left edge of the heatmap. Clustering of ABT-888’s EN genes places ARF4, C20ORF43, GANLT10 and CCND1 together with ABT-888’s GI50 profile, with greater sensitivity associated with relative under expression of these genes. GSEA for these 28 EN genes finds only the CELL_PROLIFERATION (GO:0008283) pathway (P<4.50e-7). Noteworthy in this heatmap is the correspondence of ABT-888 sensitivity with under expression of ARF4. Recently Woo et al. [29] identified ADP-ribosylation factor 4 (ARF4) as a suppressor of N-(4-hydroxyphenyl) retinamide-induced cell death. Their yeast-based functional screening for inhibitors of Bcl-2-associated X protein (Bax)-induced cell death, identified ADP-ribosylation factor 4 (ARF4) as a novel anti-apoptotic gene. Their result is consistent with the minimal EN heatmap whereby under expression of ARF4 would mitigate its anti-apoptotic role, perhaps contributing to enhanced ABT-888 sensitivity and hypothesizing ARF4 as a potential biomarker for ABT-888 sensitivity.

**Fig 5:**
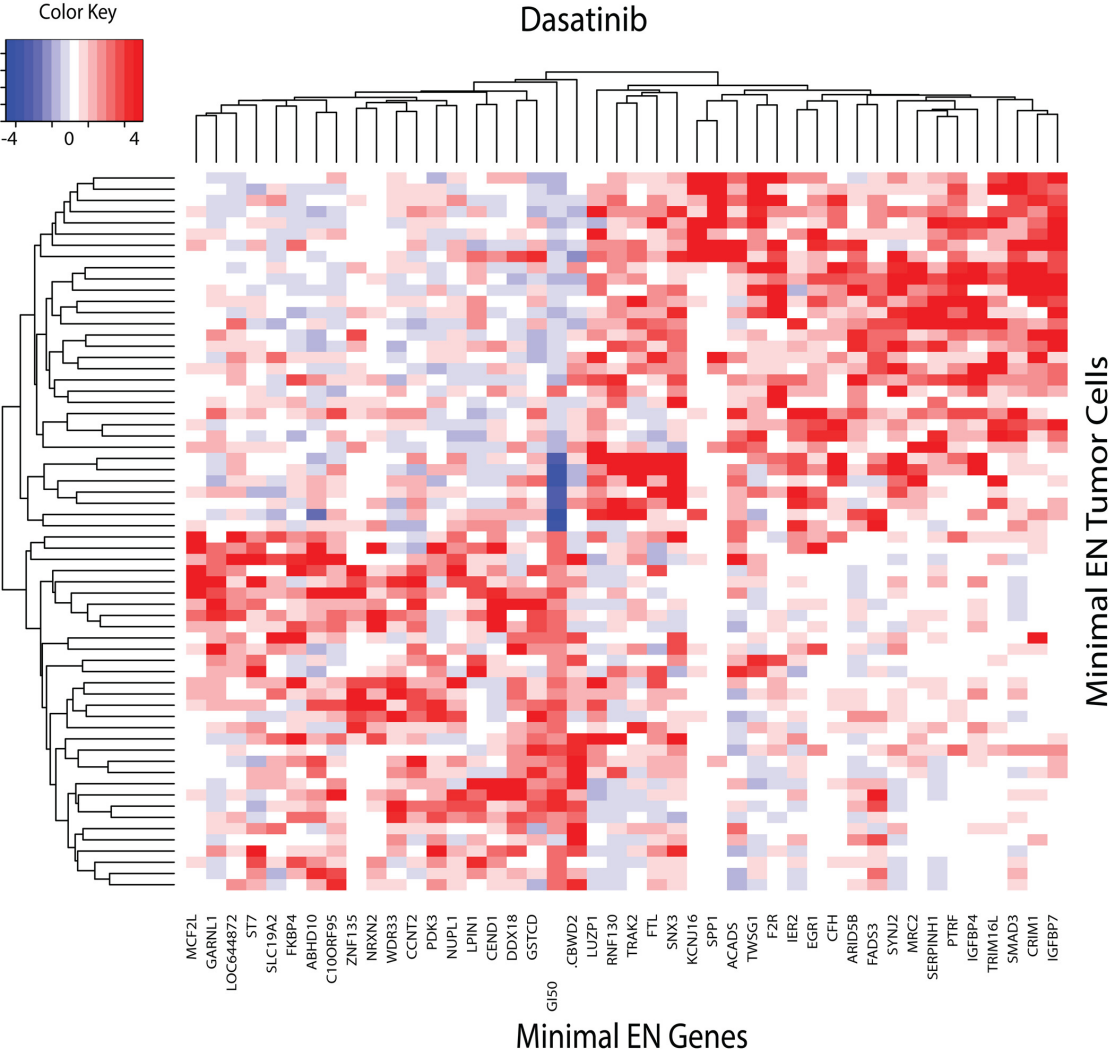
Heatmap for minimal EN GEs (x-axis) versus the upper and lower 10^th^ percentile of sensitive and resistant tumor cells (y-axis) for dasatinib. Relative over and under expression is denoted by red and blue, respectively. GI50 values for dasatinib are imbedded in the heatmap, located near the center, with its label offset towards the bottom from the EN gene labels. GI50 values for sensitive and resistant tumor cells are indicated by blue and red colors, respectively.

The dasatinib minimal EN heatmap (**Fig 5**) reveals a distinct set of seven tumor cells, all lymphomas(MEG-01:908126:CML:blood, LAMA-84:907783:CML:blood, EM-2:906855:CML:blood, CTV-1:753548:AML:blood, EoL-1-cell:906856:haematopoietic_neoplasm_other:blood, BV-173:910710:CML:blood and BL-70:910707:Burkitt_lymphoma:blood), that exhibit the greatest sensitivity to dasatinib, with its GI50 indicated by the dark blue vertical band in the central portion of the image. GSEA for the minimal EN genes associated with dasatinib sensitivity are listed in **Table D**. While the CASPASE and APOPTOTIC pathways would be expected, as found in the CGP report[7], three of these GSEA pathways involve molecular traffic. Previous studies have demonstrated that SRC Family Kinases (SFKs) influence nuclear EGFR translocation and expression[30]. More recently Brand et al.[31] report that inhibition of nuclear EGFR translocation leads to a subsequent accumulation of EGFR on the plasma membrane, which has been observed to enhance sensitivity of triple negative breast cancer cells to cetuximab [32]. The studies of Brand et al. [31] suggest that targeting both the nuclear EGFR signaling pathway, through the inhibition of its nuclear transport, and the classical EGFR signaling pathway, with cetuximab, may be a viable approach for the treatment of patients with triple negative breast cancer. These results follow earlier studies by Kahn et al.[33] supporting the role of transport in the effectiveness of SRC targeting agents. EGFR down-regulation was found to involve trafficking of activated receptor molecules from the plasma membrane, through clathrin-coated pits, and into the cell for lysosomal degradation, where oxidative stress was found to play a role in transporting EGFR to a perinuclear location, where it is not degraded and remains active. Thus, drugs affecting tumor growth may involve factors that activate EGFR and factors that modulate EGFR trafficking from the plasma membrane to sites within the cell.

### Global analysis of CN and MUT in minimal EN GEs

The previous sections illustrate the analysis of minimal EN genes, directly, to identify GEs that correlate with each drug’s GI50, and indirectly, via GSEA, to propose testable hypotheses about pathways involved in drug response. This approach can be extended to provide a global analysis of the CGP data describing MUTs and CNs that potentially play a role in drug response. The results for significant gene MUTs will be discussed first. Thirty-three percent of the 85 genes with mutation data (28 /85 MUTs = 0.33) for 41 of the 138 drugs screened achieved B-H adjusted statistical significance. **Table E** summarizes the total counts for a gene MUT appearing in these 41 drugs, as well as the total counts for a drug appearing in these 28 MUTs. Summarizing these results, a maximum of 24 drugs had CDKN2a.p14_MUT, while only single MUTs were found for SMAD4, MDM2, EGFR and CDH1. Drugs AZD7762, CEP-701 and camptothecin had greater than 8 significant gene MUTs, while only single MUTs were found for RDEA119, PD-173074, PD-0325901, NU-7441, Embelin, CI-1040, Bexarotene, AZD6244, AP-24534 and ABT-263. These results indicate the existence of shared MUTs across drugs from different MOA classes and the possibility for combinations of MUTs playing roles in drug response.

**Fig 6:**
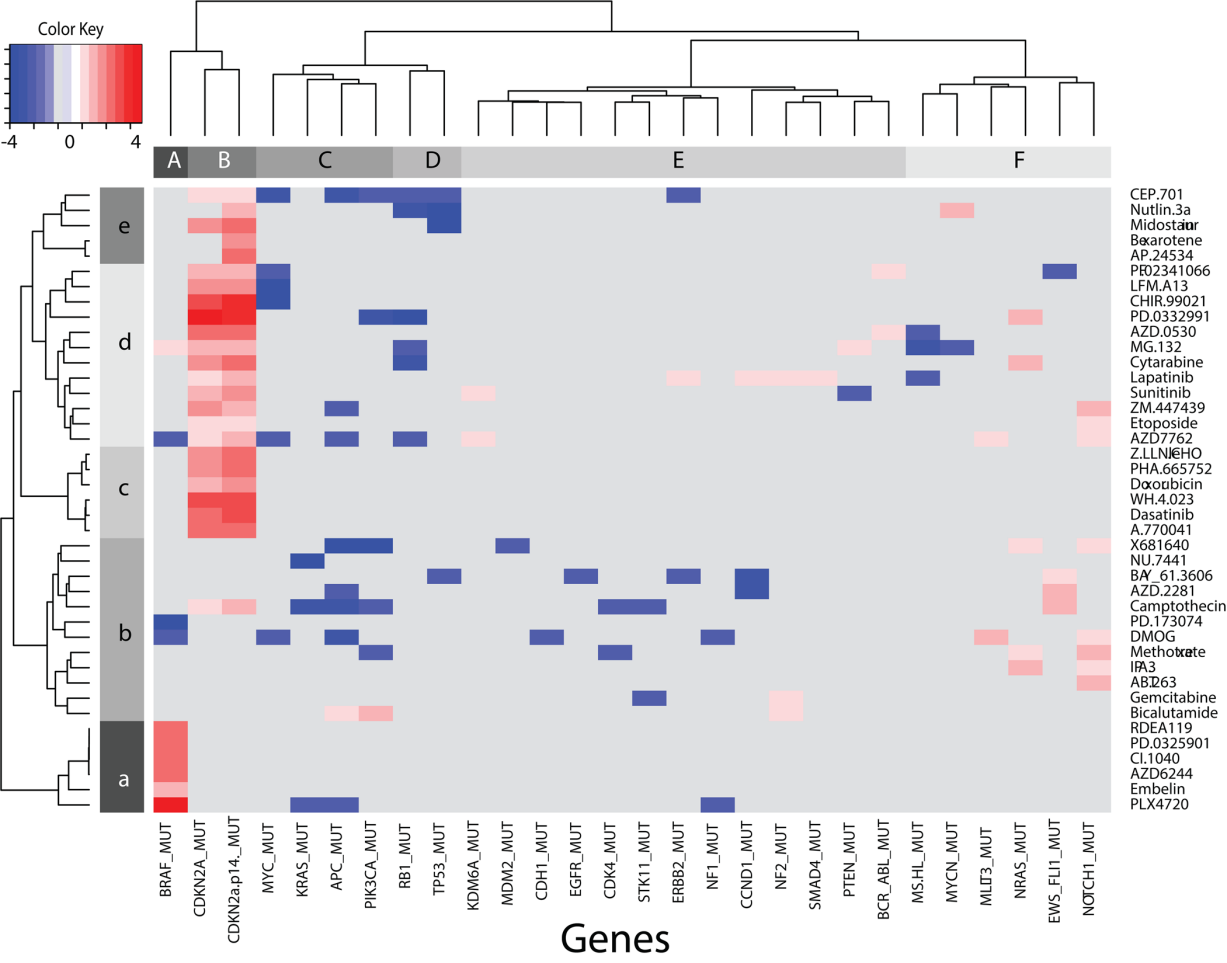
Heatmap for drug-MUT combinations achieving statistical significance (B-H q-value = 0.1). The metric used to cluster this data is based on the t-statistic generated from the Student’s t-test, where shades of red indicate cases where the gene MUT appears predominately within the sensitive tumor cells, while shades of blue find the MUT existing predominately within the resistant tumor cells. Bars at the base of the top and left dendrograms identify sub-clade members (lower-case letters a-e correspond to drugs and upper-case letters A-F correspond to MUTs).

**Fig 6** displays the heatmap for the drug-MUT combinations achieving B-H adjusted statistical significance across all compounds tested in the CGP database. The metric used for clustering this data is based on the t-statistic generated from the Student’s t-test, where shades of red or blue indicate cases where the MUT appears predominately within the sensitive or insensitive tumor cells, respectively. To summarize these results, the row (drugs) and column (MUTs) dendrograms in **Fig 6** have been collapsed into 5 and 6 meta-clades, respectively, labelled a-e and A-F, appearing as bars at the base of each dendrogram. Clustering of the drugs (i.e. rows) will be considered first. The row dendrogram at the left edge divides the drugs into a lower branch (row-clades a and b) and an upper branch (row-clades c, d and e); distinguished mainly by the presence or absence of CDKN2a_MUT and CDKN2a.p14_MUT. The upper branch is further divided into sub-branches that either lack other significant MUTs (row-clade c) or have at least one significant MUT (row-clades d and e). The lower branch of drugs, lacking CDKN2a_MUT or CDKN2a.p14_MUT, is divided into those with BRAF_MUT (row-clade a) and without BRAF_MUT (row-clade b). Row-clade a is comprised of tumor cells sensitive to six drugs (PD-0325901, RDEA119, CI-1040, AZD6244, Embelin and PLX4720). Row-clade b, which lacks either BRAF_MUT or CDKN2a.p14_MUT, is comprised of twelve drugs (681640, NU-7441, BAY_61.3606, AZD-2281, camptothecin, PD-173074, DMOG, methotrexate, IPA-3, ABT-263, gemcitabine and bicalutamide) and consists of a mixture of MUTs with significant differences between sensitive and resistant tumor cells. Significant MUTs in row-clade b appear primarily at the right most columns in **Fig 6** and are associated with MLLT3_MUT, NRAS_MUT, EWS-FLI1_MUT and NOTCH_1_MUT (column-clade F in upper dendrogram). Consistent with the CGP results [7], camptothecin sensitive tumor cells have EWS-FLI1_MUT, a feature also shared by tumor cells sensitive to AZD-2281 and BAY-61-3606 (row-clade b and column-clade F). Within column clade F, NOTCH-1_MUT appears to be important for sensitivity to ZM_447439, etoposide and AZD_7762 (row-clade d), and 681640, DMOG, methotrexate, IPA-3 and ABT-263 (row-clade b). Also consistent with the CGP results [7] is lapatinib’s (row-clade d) sensitivity to tumor cells with ERBB2_MUT (column-clade E), which, according to these results, is also accompanied by MUTs in CCND1, NF2 and SMAD4. The importance of these co-existing mutations has been previously discussed in the CGP report[7], where enhanced sensitivity to ERBB2/EGFR targeting agents appears to be related to elevated EGFR expression in tumor cells with SMAD4_MUT. The results of **Fig 6** suggest potentially important co-mutations for a majority of the drugs achieving B-H adjusted statistical significance. Overall 60% (9 of 15 genes) of the gene MUTs independently identified in the CGP dataset by Ding et al.[34] appear as significant MUTs in this analysis.

The column dendrogram at the top edge of **Fig 6** divides the MUT genes into those found predominately in the sensitive tumor cells (column-clades A, B and in the right-most branch of column clade F, with the middle clades (column-clades C,D and E) are associated with MUTs existing primarily in resistant tumor cells. Noteworthy is the co-clustering of mutated genes MYC, KRAS, APC and PIK3CA in column-clade C and the co-clustering of RB1 and TP53 in column-clade D. The co-existence of PIK3CA_MUT and KRAS_MUT has been observed in non-small cell lung cancer [35]. Co-existing APC_MUT and oncogenic KRAS_MUT have been found to enhance Wnt signaling [36] and are associated with specific chromosomal aberrations in colorectal adenocarcinomas [37]. In addition, an independent analysis of combinatorial patterns of somatic gene mutations in the CGP data also finds the co-existence of TP53_MUT with RB1_MUT [38]. The general observation made from **Fig 6** is that while selected gene MUTs in sensitive tumor cells may contribute to drug sensitivity, of potentially equal importance may be the contribution of co-mutated genes to drug resistance for many of these compounds (as described above with ERBB2_MUT and SMAD4_MUT for lapatinib sensitivity). To further emphasize this finding, the case of PLX4720 can be examined. PLX4720 appears as the bottom row of **Fig 6** (row-clade a), with MUTs in sensitive tumor cells for BRAF_MUT (column-clade A) and resistant tumor cells for MUTs in KRAS, APC and NF1 (column-clades C and E). Ding et al. [34] support these results with the finding of somatic mutations in primary lung adenocarcinoma for several tumor suppressor genes involved in other cancers, including APC and NF1. Ahlquist et al. [39] find that RAS signaling in colorectal carcinomas originates through alterations of RAS, RAF, NF1, and/or RASSF1A. Consistent with the roles of MUTs in the response of PLX4720 for lung and colon cancer, analysis of the minimal EN model for PLX4720 finds that 20% of its resistant cells to be derived from lung cancers and 17% originating from colon cancers. Other reports find melanomas with BRAF_MUT are sensitive to PLX4720. Consistent with this observation, the minimal EN model for PLX4720 finds that 36% of PLX4720 sensitive tumor cells are derived from malignant melanoma. Collectively, and consistent with the CGP report[7], this global analysis supports combinations of MUTs to be potentially exploitable as predictive biomarkers of sensitive and resistant drug responses.

**Fig 7:**
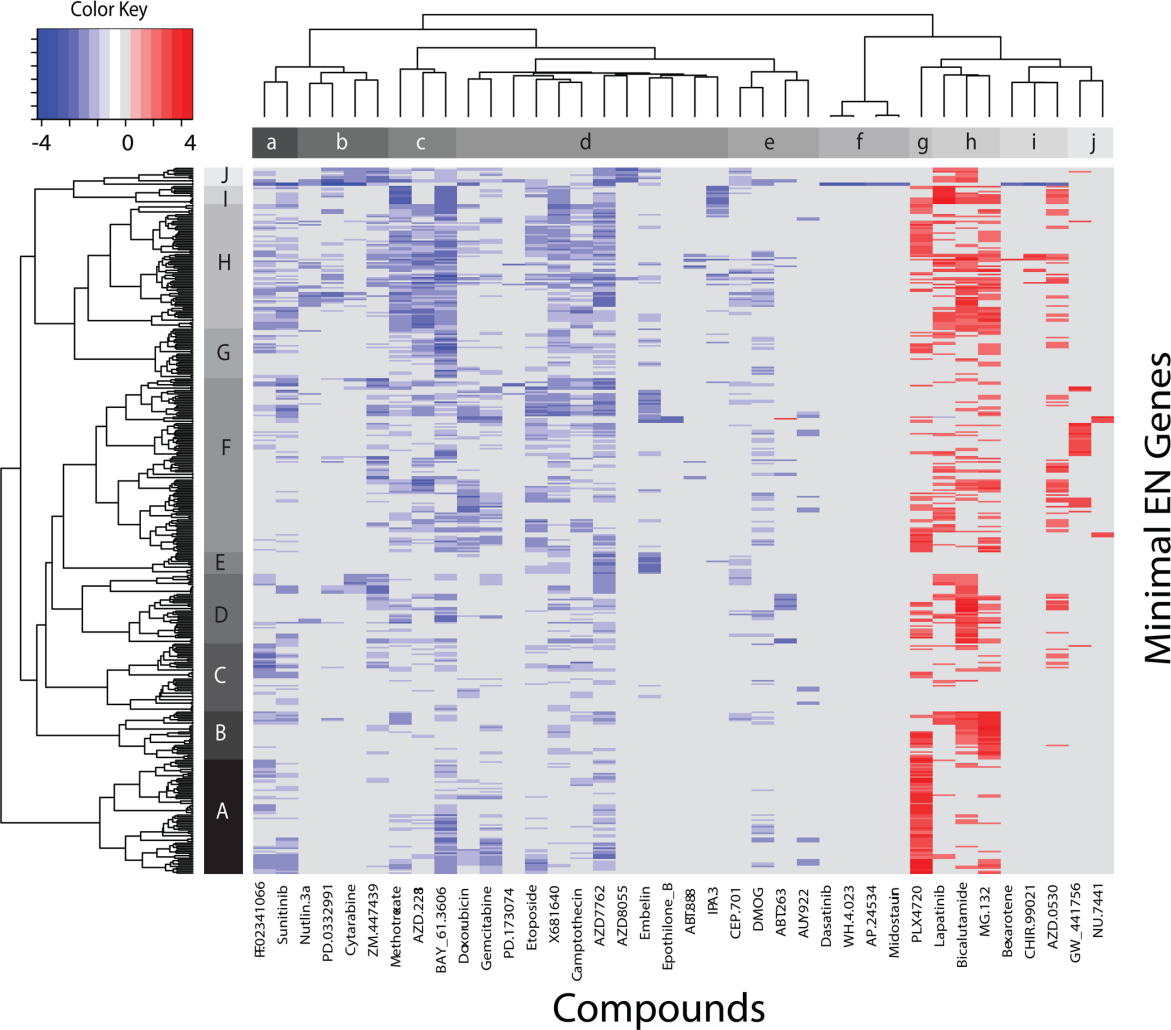
Heatmap for genes (y-axis) with significantly different CN changes between sensitive and resistant tumor cells for the minimal EN models of each drug versus drugs (x-axis). These results find 426 genes with significant CN differences for only 38 drugs (B-H adjusted q value = 0.1). Blue and red colors indicate CN changes occurring within resistant and sensitive tumor cell populations, respectively. Bars adjacent to left and top dendrograms identify sub-clades members, respectively (Upper-case A-J for gene_CN and lower-case letters a-j for drugs).

The heatmap in **Fig 7** displays the results from the analysis of CN changes that are significantly different between sensitive and resistant tumor cells for the minimal EN models of each drug. These results find 426 genes, from a total of 433 CNs measured in the CGP dataset, with significant CN differences for only 38 drugs (B-H adjusted q value = 0.1). Remarkably, nearly all of the measured CNs achieve statistical significance for at least one of these 38 drugs. As with the MUTs results, the CN results will be summarized by referencing the collapsed clades shown as bars at the base of each dendrogram in **Fig 7**. The genes in row-clades A through J are listed in **Table F**. Thirty-three of these drugs are shared with the MUT results discussed above (33/41 = 0.8, **Fig 6**). Inspection of **Fig 7** reveals three distinctive features of these global CN results. First, significant CN differences are nearly completely segregated between those occurring primarily in the sensitive tumor cells (shades of red, column-clades g, h, i and j) and those occurring primarily in the resistant tumor cells (shades of blue, column-clades a, b, c, d, e and f). Second, CN changes significantly greater for the sensitive tumor cells exist only for

1. PLX4720:BRAF (column-clade g),
2. Lapatinib:ERBB2, Bicalutamide:Androgen receptor, MG-132:Proteasome (column-clade h),
3. Bexarotene:Retinoic acid receptor, CHIR-99021:GSK3B, AZD-0530:SRC,ABL1 (column-clade i)
4. GW_441756:NTRK1, NU-7441:DNAPK (column-clade j).

Third, the remaining 29 drugs have CN changes primarily within resistant tumor cells.

GSEA of these 426 genes with significant CN differences between sensitive and resistant tumor cells strongly identifies pathways involved in cancer, either in their metabolic or biosynthetic nuclear processes, DNA binding or transcription. **Table G** summarizes the top 10 GSEA processes using these 426 genes. These results are consistent with what would be expected for actively dividing tumor cells.

The number of genes with CN differences for each drug (i.e. number of non-zero cells for each column in **Fig 7**) finds as the top four drugs; PLX4720 (n = 188 CN differences), bicaluamide (n = 177), MG-132 (n = 174) and lapatinib (n = 124). The following sections will expand on the results for 3 of these drugs.

PLX4720’s EN genes have the most significant CN differences, based on their t-statistic, ranking KIAA1549_CN, BRAF_CN and CREB3L2_CN at the top of the list. The occurrence of BRAF_CN as the second ranking member in this set is consistent with strong literature support for the PLX4720-related compound vemurafenib targeting BRAF in the treatment of melanoma[40]. The top ranked member, KIAA1549_CN, represents a novel finding. The protein encoded by KIAA1549 has been found to be fused to the BRAF oncogene in many cases of pilocytic astrocytoma. The fusion results from 2Mb tandem duplications at 7q34 (provided by RefSeq, Oct 2012). Less is known about the third ranking CN gene, CREB3L2 (cAMP responsive element binding protein) in PLX4720 sensitivity. The recent discovery of a novel CREB3L2-PPARgamma fusion mutation in thyroid carcinoma with t(3;7)(p25;q34), demonstrated that a family of somatic PPARgamma fusion mutations exist in thyroid cancer [41]. Four of the 26 CGP tumor cells exhibiting sensitivity to PLX4720 are thyroid in origin. The extent to which CN changes in CREB3L2 influence PLX4720 sensitivity will require further study. Alternatively, studies have shown that the PLX4270 analog, vemurafenib, is a potent inducer of endoplasmic reticulum stress-mediated apoptosis[42]. CREB3L2, also known as BBF2H7, is an ER-resident transmembrane protein with the bZIP domain in the cytoplasmic portion that is cleaved at the membrane in response to ER stress. The cleaved fragments of BBF2H7 translocate into the nucleus and can bind directly to cyclic AMP-responsive element sites to activate transcription of target genes[43]. Notably, BRAF and CREB3L2 are both in the KEGG pathway for prostate cancer, thus raising the possibility that PLX4720 may also play a role in interfering with CREB3L2 binding to DNA and impacting transcription, possibly in prostate cancer. Only 5 tumor cells of prostate origin are included in the CGP data.

Lapatinib’s EN genes (column-clade j, **Fig 7**) with CN changes having the greatest statistical significance are all located in row-clade I and consist of HOX family members HOXA9, HIXA11 and HOXA13. Gilbert et al.[44] report that HOXA9 regulates BRCA1 expression to modulate a human breast tumor phenotype. Their study identified homeobox A9 (HOXA9) as a gene frequently down regulated in human breast cancers, and noted that reduced HOXA9 transcript levels are associated with tumor aggression, metastasis, and patient mortality. Restoring HOXA9 expression repressed growth and survival and inhibited the malignant phenotype of breast cancer cells in culture and in a xenograft mouse model. Gilbert et al.[44] speculate that HOXA9 restricts breast tumor aggression by modulating expression of the tumor suppressor gene BRCA1. The finding here supports the use of HOXA9_CN as a predictor of lapatinib sensitivity. Increases in EGFR_CN for lapatinib sensitive tumor cells are also located in row clade I, placing 9^th^ in the gene rankings, based on their t-statistic. EGFR_CN has been proposed as a predictor of lapatinib sensitivity in HER2-positive metastatic breast cancer [45].

Bicalutamide (target:androgen receptor) appears in column-clade j (**Fig 7**), with EN genes having the most significant CN changes all located in row-clade D. The genes with the most significant CN changes are TSC1, CD74, APC and NOTCH-1. GSEA results place these genes in pathways associated with NEGATIVE REGULATION OF CELLULAR PROCESSES and CELL PROLIFERATION. These 4 genes have indirect and direct associations with autophagy. For the case of TSC1, the interaction is indirect. Androgen deprivation, or treatment with the anti-androgen bicalutamide, has been reported to promote autophagy[46]. This effect could be dramatically reduced after depletion of Atg5 and Beclin-1, two canonical autophagy genes, and was associated with inhibition of the androgen-induced mTOR pathway, with a significant increase in bicalutamide-induced cell death. The mTOR complex functions as a nutrient/energy/redox sensor and controller of protein synthesis. Most of the variables required for protein synthesis affect mTOR activation by interacting with the GTPase activating TSC1/TSC2 protein complex. Elevated CN changes in TSC1 support its role in autophagy-induced cell death. The second most significant gene with CN differences, CD74, represents another indirect association with the androgen receptor. Macrophage migration inhibitory factor (MIF: a pluripotent cytokine with important roles in many cellular processes including cell proliferation, angiogenesis, and tumorigenesis[47]) binds to CD74 to transduce signals for the secretion of TNF-α as well as play a role in cell autophagy. Increased CD74_CN may also play a role in bicalumide-induced cell death. Involvement of the 3^rd^ and 4^th^ most significant genes, APC and NOTCH-1, in autophagy, as reported by Shimobayashi and Hall [48], find emerging evidence into the control of mTOR by other pathways such as Hippo, WNT and NOTCH signaling. Notably, WNT activates mTOR through loss of APC, whereas NOTCH signaling may affect WNT signaling as an indirect regulator of autophagy[49]. These results identify a connection between bicaluamide sensitivity and CN changes for genes involved in autophagy.

A noteworthy feature of the heatmap in **Fig 7** is the complete lack of genes with significant CN changes for the 4 drugs in column clade f (Dasatinib, WH-4_023, AP_24534 and Midostaurin), with the exception of CN increases in CDKN2A and CDKN2a.p14, only for resistant tumor cells. This result hypothesizes a link between resistance to these drugs and increased CDKN2A_CNs.

### GSEA analysis of significant MUTs and CNs

**Fig 8:**
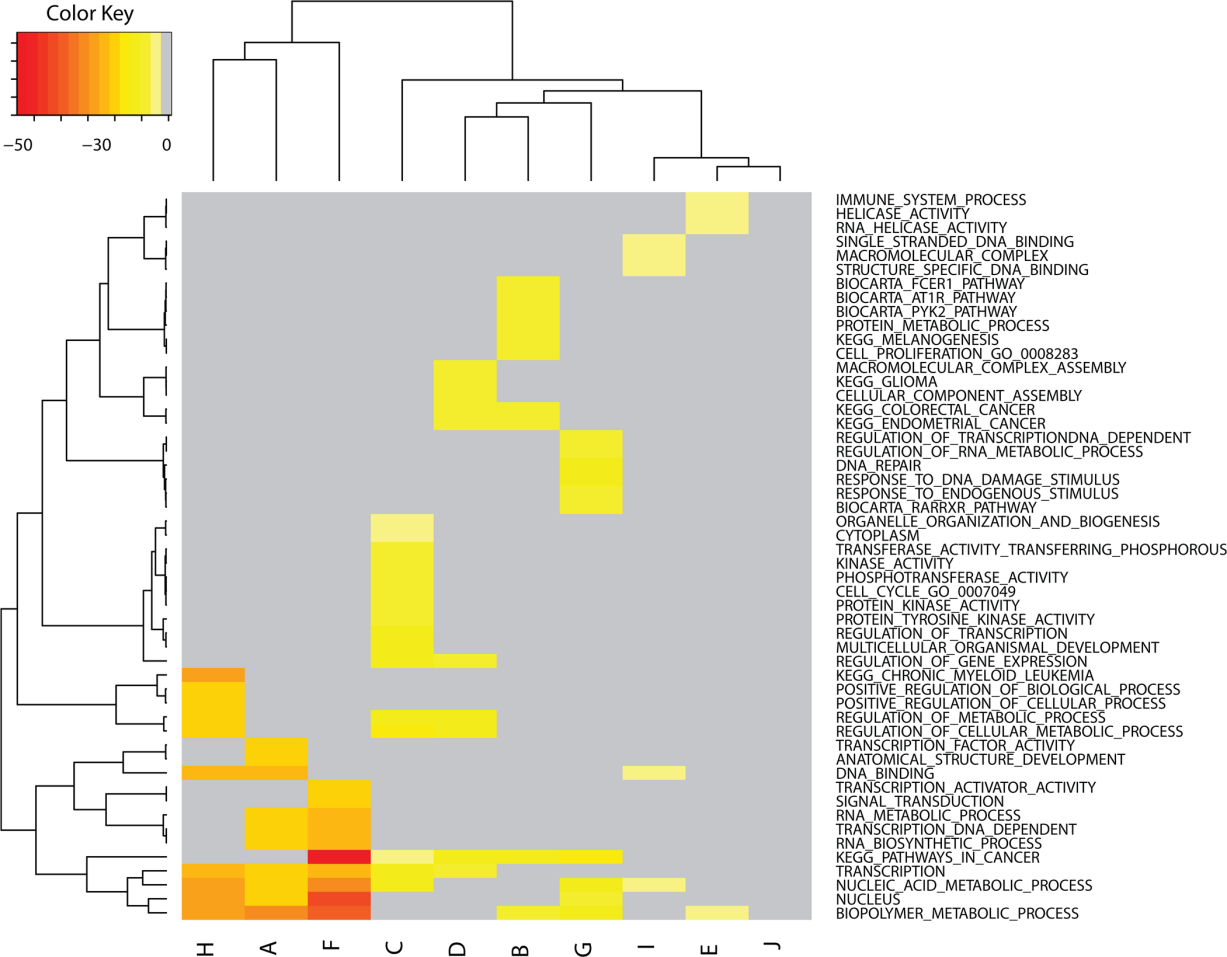
Heatmap using GSEA results for genes within row meta-clades A-J of Fig 7. The metric used for clustering is the log of the FDR q-values determined from each GSEA result. GSEA scores are represented spectrally from most (red) to least (yellow) significance. Only the top 10 scoring GSEA pathways are selected from genes derived from each of the 10 (A-J) row-clades of **Fig 7**.

Genes within row-clades A-J of **Fig 7** can be examined collectively by GSEA to identify associations between CN changes and biological pathways. **Fig 8** displays the heatmap for the 52 most significant GSEA pathways derived from CN genes selected from row-clades A-J (cf. **Fig 7)**. The metric used for clustering is the log of the FDR q-values determined from each GSEA result. The tabular listing of the heatmap values appears in **Table H**. Column-clades H, A and F of **Fig 8** have the most significant FDR q-values among this set and are clustered as the first three columns at the left. The CN genes for these clades identify pathways associated primarily with

1. nucleic-acid regulation of biological processes (NUCLEUS, NUCLEIC_ACID_METABOLIC_PROCESS, TRANSCRIPTION, RNA_BIOSYNTHETIC_PROCESS, TRANSCRIPTION_DNA_DEPENDENT, RNA_METABOLIC_PROCESS, TRANSCRIPTION_ACTIVATOR_ACTIVITY, DNA_BINDING, TRANSCRIPTION_FACTOR_ACTIVITY),
2. cellular metabolism (REGULATION_OF_CELLULAR_METABOLIC_PROCESS, REGULATION_OF_METABOLIC_PROCESS, POSITIVE_REGULATION_OF_CELLULAR_PROCESS, POSITIVE_REGULATION_OF_BIOLOGICAL_PROCESS)
3. signal transduction (SIGNAL_TRANSDUCTION).

These results are consistent with the previous GSEA results for the complete set of 426 genes with significant CN differences between sensitive and resistant tumor cells.

Column-clade C in **Fig 8** represents a unique sub-clade, with genes identifying GSEA pathways for

1. kinase activity (PROTEIN_TYROSINE_KINASE_ACTIVITY, PROTEIN_KINASE_ACTIVITY, PHOSPHOTRANSFERASE_ACTIVITY, KINASE_ACTIVITY, TRANSFERASE_ACTIVITY_TRANSFERRING_PHOSPHORUS_CONTAINING_GROUPS),
2. regulation of transcription (TRANSCRIPTION, TRANSCRIPTION_DNA_DEPENDENT, TRANSCRIPTION_ACTIVATOR_ACTIVITY)
3. regulation of gene expression (REGULATION_OF_GENE_EXPRESSION).

Inspection of **Fig 8** finds that CN changes for the genes in **Fig 7,** row-clade C, affect resistance (blue shaded t-statistic) and sensitivity (red shaded t-statistic) depending on the drug. In particular, **Fig 7,** row-clade C, column-clades g and h represent CN increases involved in drug sensitivity for PLX4720 and lapatinib. The most frequently shared genes in the GSEA pathways associated with column-clade C of **Fig 8** are KIT, KDR, PDGFR1 and PDGFRA. The combined analysis of kinase genes with statistically significant CN differences and their associated GSEA pathways support a connection between kinase CN and tumor cell sensitivity for drugs that target kinase activity.

**Fig 8** column-clades D, B, G, and column-clades I, E, J are grouped as neighboring sub-clades. Column-clade E, I and G are associated with helicase activity, nucleic acid binding and DNA damage/repair, respectively. Column-clade B is associated with BIOCARTA pathways for FCER1, AT1R and PYK2; each with roles in PKC-dependent Ca+ signaling. Noteworthy is the absence of any significant GSEA pathways for column-clade J, which includes the CDKN2A and CDKN2A.p14 genes.

## Discussion

**Fig 9:**
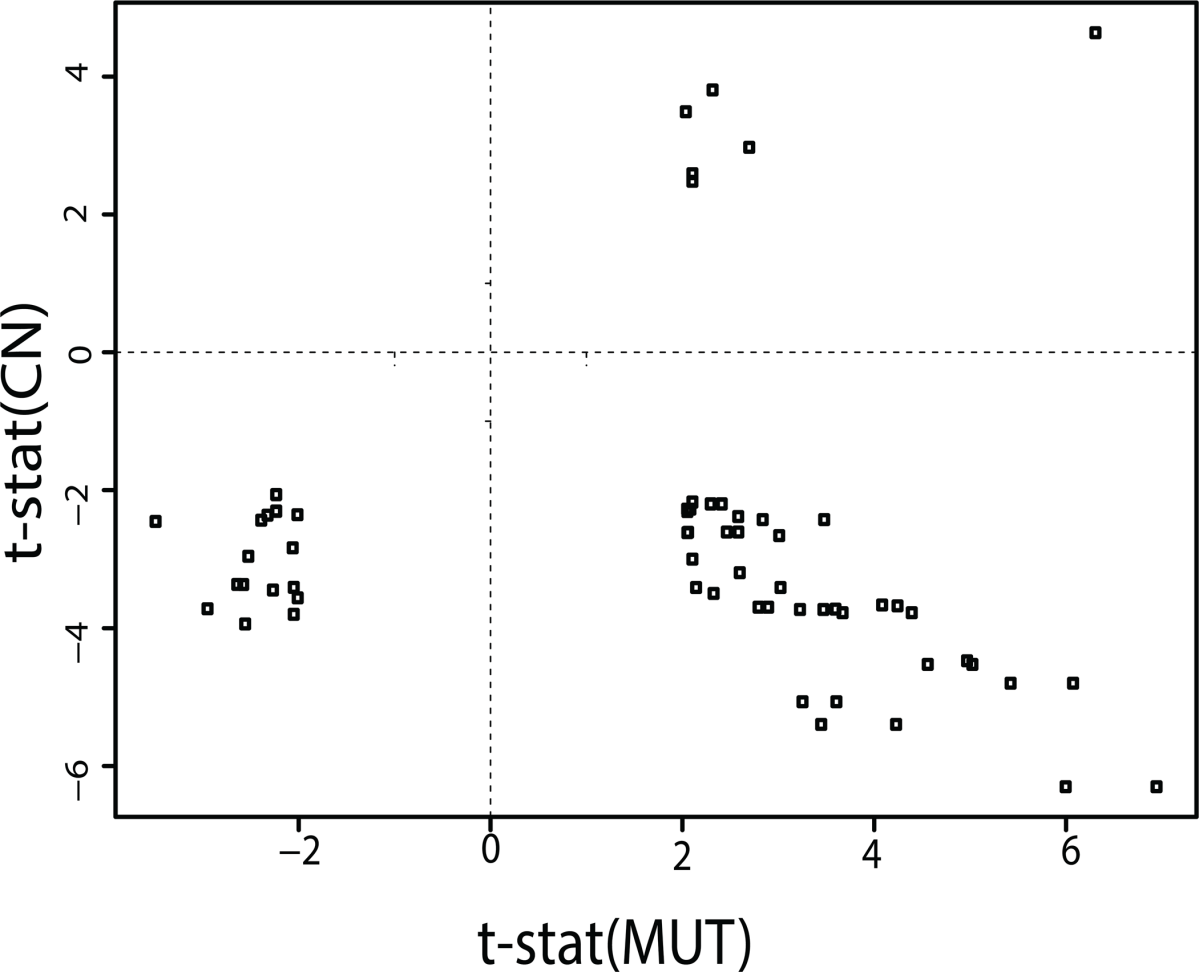
Plots of the t-statistic for genes sharing statistically significant CN changes and MUTs between sensitive (t-stat >0) and resistant (t-stat<0) tumor cells for their minimal EN model. The table of tstat(CN) and tstat(MUT) values is listed in **Table I**.

The results of this analysis find that minimal EN GEs, MUTs and CNs can potentially be used as biomarkers of drug response. Choosing which biomarker provides the best indicator of drug response represents a major challenge to their effective use. Evident from the global analysis is that GE, MUT or CN, separately or in combination, may appear both as indicators of sensitivity or resistance, and can do so as a result of relatively high or low values, depending on the test drug. To illustrate this point, **Fig 9** plots the t-statistic for 64 cases of minimal EN genes that collectively share statistically significant CN changes and MUTs between sensitive (t-stat >0) and resistant (t-stat<0) tumor cells for genes in their minimal EN model. These results, representing only 17 genes and 28 compounds, can be examined for cases where MUT and CN changes track together versus in opposite directions. Twenty-three cases exist where statistically significant CN and MUT results co-exist within the sensitive tumor cells (t-stat >0, n = 6, upper right quadrant) or resistant (t-stat<0, n = 17, lower left quadrant) tumor cells. The lower-left quadrant is composed of MUT and CN changes for BRAF, MYC, KRAS, APC, PIK3CA, MDM2, EGFR, CDK4, ERBB2, CCND1. The upper-right quadrant is composed of MUT and CN changes for BRAF, APC, PIK3CA, ERBB2, CCND1. The co-occurrence of BRAF, APC, PIK3CA, ERBB2 and CCND1 in both quadrants indicates that their genomic changes have roles in sensitivity and resistance, at least for the CGP drugs. The drugs associated with these two quadrants are mutually exclusive, to suggest that apparent associations between MUT and CN changes and drug response most likely represent distinct biological processes. The majority of cases (n = 42, lower-right quadrant) find a discordance between CN and MUT results such that MUTs appear in the sensitive tumor cells while CN changes appear in the resistant tumor cells. The origins of this observation, and its consequences, will require further study. Nonetheless, these results support relatively unique associations between a drug’s response and MUT or CN status. In support of these findings, examination of the raw GDS-COSIMC data, regardless of selecting subsets based on resistance or sensitivity, finds that fifty-two cases exist where MUT and CN changes exist for a gene. One-third of these cases (n = 17) are significantly (p<0.05) anti-correlated while the remaining 35 cases are significantly positively correlated. The 17 anti-correlated genes all appear in the lower right quadrant of **Fig 9**. These results suggest that the general trend of both concordance and discordance for MUT and CN changes observed above exists within the raw data.

Genes that co-exist either as MUTs or CNs and their minimal EN GEs may offer a degree of redundancy that strengthens their individual utility as biomarkers. **Table 3** lists the 28 cases (15 drugs and 21 genes) where a gene co-exists within each drug’s minimal EN model and has a statistically significant difference in their MUT or CN values between sensitive and resistant tumor cells. Cases of CN changes dominate these results, most likely because of the larger numbers of CN changes versus MUTs within the CGP dataset. These results suggest that the co-existence of EN GEs, MUTs and CNs may offer strategies for prioritizing drug selection based jointly on these three measurements. Notable in **Table 3** is the occurrence of only 4 instances where a gene is present in the minimal EN model and has a statistically significant difference in both the gene’s MUT and CN values;

1. AZD-2281:CCND1
2. Cytarabine, Nutlin-3a and PD-0332991:CDKN2A
3. Lapatinib:ERBB2
4. PF-02341066:BRAF.

**Table 3:** CN and MUT values that are significantly (B-H q value of 0.1) different between resistant and sensitive tumor cells. Column 1: Drug, column 2:gene_CN, column 3:gene_MUT, column 4:Drug Target.

Drug	CN	MUT	Target
AZD-2281	CCND1	CCND1	PARP1/2
AZD-2281	IDH1	-	PARP1/2
AZD-2281	IL21R	-	PARP1/2
AZD-2281	LMO2	-	PARP1/2
AZD7762	CDK6	-	CHK1/2
AZD7762	FLI1	-	CHK1/2
AZD7762	PDE4DIP	-	CHK1/2
BAY_61-3606	KLK2	-	SYK
BAY_61-3606	-	TP53	SYK
Camptothecin	HOXD11	-	TOP1
CEP-701	CBL	-	FLT3, JAK2, NTRK1, RET
CEP-701	-	ERBB2	FLT3, JAK2, NTRK1, RET
Cytarabine	CDKN2A	CDKN2A	Ara-Cytidine
Etoposide	MAF	-	TOP2
Gemcitabine	BCL10	-	DNA replication
Gemcitabine	JAK3	-	DNA replication
Gemcitabine	PDE4DIP	-	DNA replication
Lapatinib	ERBB2	ERBB2	EGFR, ERBB2
Methotrexate	IKZF1	-	DHFR
Methotrexate	LMO2	-	DHFR
Nutlin-3a	CDKN2A	CDKN2A	MDM2
PD-0332991	CDKN2A	CDKN2A	CDK4/6
PD-0332991	-	RB1	CDK4/6
PF-02341066	BRAF	BRAF	MET, ALK
PLX4720	GAS7	-	BRAF
PLX4720	IDH1	-	BRAF
ZM-447439	PPARG	-	AURKB
ZM-447439	WT1	-	AURKB

Dashes represent absence of statistical significance. Listing is sorted by drug target.

This observation is consistent with the above noted importance of MUTs and CNs in BRAF, ERBB2, CDKN2A and CCND1 for these drugs.

### ROC analysis of CGP GEs as predictive of CCLE drug response

**Fig 10:**
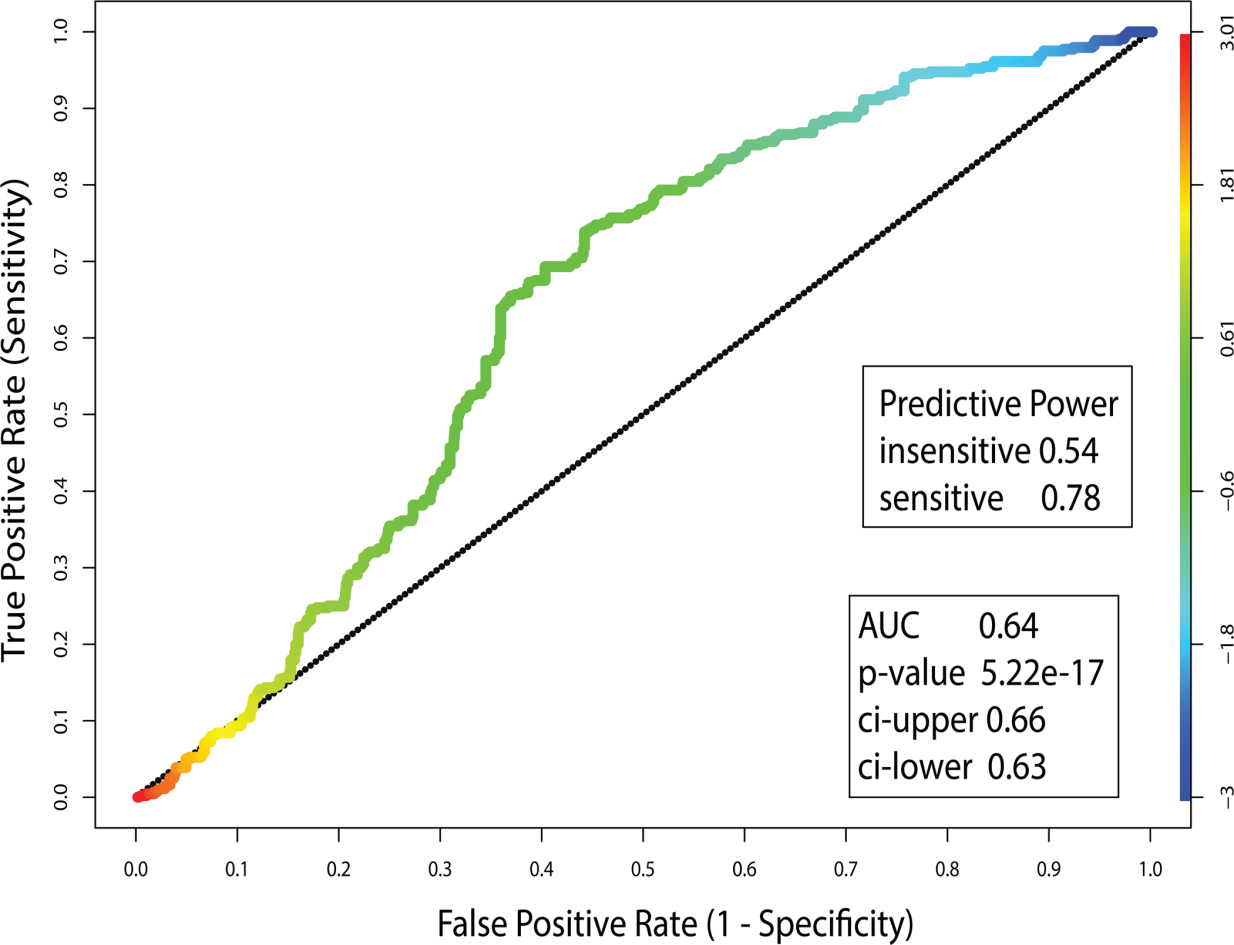
Receiver operator curve (ROC) based on using the CGP minimal EN GEs to select tumor cells with the most similar GEs. GI50 responses are selected from the top 5^**th**^ percentile of CCLE tumor cells with GEs that best match the CGP EN GEs. ROC is spectrally color coded from red (resistant) to blue (sensitive) tumor cells.

The CCLE dataset can be used to assess whether minimal EN GEs developed for the CGP set of drugs may be used separately to select for other tumor cells with matching GEs; inferring that tumor cells with the best matching GEs might also share the test drug’s response. Failure to achieve any success with this method could influence future applications of this approach. However, moderate success may offer motivation for devising more optimal steps for achieving favorable outcomes with this approach. The CCLE dataset (24 drugs tested against 479 tumor cells) shares 16 drugs with the CGP datasets. A strong positive correlation was reported in both CN and GEs between the CGP and CCLE datasets[8]. Using the CGP-derived minimal EN model for each of the 16 matching drugs, gene expressions between these two datasets will be compared (using their mean squared error, MSE) and used to rank the complete set of CCLE’s tumor cells. In order for the ‘test’ biomarker to have predictive utility, MSE scores must correctly rank a CCLE tumor cell’s drug response within the top (sensitive) or bottom (resistant) of all CCLE tumor cells. The result of this analysis selects only the top 5^th^ percentile of MSE scores for the CCLE tumor cells. It is noteworthy to re-emphasize that the minimal EN model uses gene expressions to predict GI50. Thus sensitivity and resistance is an integral part of this model. Application of this test finds 1341 instances where CCLE tumor cells are found within the top 5^th^ percentile rankings for the 16 drugs jointly appearing in the CGP and CCLE datasets. **Fig 10** plots the Receiver Operator Curve (ROC) for this calculation; where true positives and true negatives correspond to cases where the CGP tumor cell’s drug response and the drug response of the best MSE-ranked CCLE tumor cells are either both sensitive or both resistant, respectively (true positive: CGP(-):CCLE(-), true negative: CGP(+):CCLE(+), where - and + indicate sensitive and resistant responses, respectively). False positives and false negatives correspond to cases where the CGP tumor cell’s drug response and the CCLE tumor cells with the best MSE scores for EN GEs do not agree (false positive; CGP(+):CCLE(-), false negative; CGP(-):CCLE(+)). The AUC of 0.64 for this ROC is highly significant (p = 5.2e-17) when compared to the null model (H0:) of no selectivity or specificity. The predictive power of 0.78 for sensitivity is an indication that within this test set of 1341 cases, ~ ¾ of the instances of tumor cell drug sensitivity in the CGP results also correspond to drug sensitivity for CCLE tumor cells having similar expressions for genes in the minimal EN model of each drug. The predictive power for resistant tumor cells is 0.54; suggesting that this approach performs slightly poorer when predicting resistance. Collectively, these results are consistent with the greatest deviation of the ROC from H0: occurring for cells having sensitivity to the test drug (blue to green portion of the color spectrum displayed along the ROC and at the left side of the image), with the least deviation from H0: occurring for drug resistant responses (yellow to red portion of the color spectrum). While these findings are preliminary, they support future applications of minimal EN GEs models, using a ‘test’ set of tumor cell’s GEs, as an *a priori* means of assessing possible drug response of an untested tumor cell.

## Summary

The results presented here support the shared conclusion of Garnett et al.[7] and Barretina et al.[8] that the CGP and CCLE datasets are information-rich, yet complicated, resources that may play pivotal roles in proposing research strategies and therapeutic decisions based on genomic biomarkers. Confirmations of many previously reported individual drug response-genomic biomarker associations are reported, with extensions that hypothesize, for example, roles of i) DNA maintenance in the sensitivity to MEK1/2 inhibitors, ii) IL-8 on the sensitivity of proteasome targeting agents, iii) autophagy on the sensitivity of mTOR targeting agents, iv) molecular trafficking on the sensitivity of SRC targeting agents and v) the HOX gene family on sensitivity to EGFR targeting agents. Global comparisons of biomarker associations across various MOA classes support existing and reveal novel perspectives for roles of combinations of GEs, MUTs and CNs in tumor cell drug sensitivity and resistance. Applications of CGP-derived genomic biomarkers to predict the drug response of CCLE tumor cells finds a highly significant ROC, with strong positive predictive powers for sensitivity and resistance. The retrospective use of GEs, MUTs and CNs as genomic biomarkers can be expected to play strong roles as hypothesis-generating engines for designing basic science studies, and when appropriately vetted as pre-therapy biomarkers for drug selection. The collective results presented here lend support to the application and further development of biomarker discoveries using a research model that applies novel data mining and analysis tools to link to drug response to various aspects of the cancer genome.

## Supporting Information

S1 File Fig A Hierarchical cluster dendrogram for the pairwise Pearson correlation coefficients of the CGP data; Fig B Heatmap of GI50 correlations for the CGP data; Fig C Glmnet EN regression output for PD-0325901; Fig D Heatmap for CGP drugs that yielded a converged EN model with 10 or more genes; Fig E: Heatmap for minimal EN model of bortezomib; Table A. Pvclust result for GI50 measures in the CGP tumor cells; **Table B.** Cluster results for EN genes; **Table C**. GSEA results for minimal EN models of PD_0325901; **Table D.** GSEA results for the minimal EN genes of dasatinib; **Table E.** Summary of counts for B-H adjusted significant MUTs; **Table F.** Clade members for row-clades A-J of main text **Fig 8**.; **Table G.** Best scoring GSEA results for global minimal EN genes.; **Table H.** Listing of -log(FDR q-values) for GSEA results using genes with significant CN changes; **Table I.** Listing of tstat values for gene MUT and CN changes for COSMIC drugs
(DOCX)
